# Less is more: inverting the paradigm across the cancer care continuum

**DOI:** 10.1016/j.eclinm.2025.103599

**Published:** 2025-10-24

**Authors:** Julien A.M. Vos, Sahar Barjesteh van Waalwijk van Doorn-Khosrovani, Hans M. Westgeest

**Affiliations:** a Department of General Practice, Amsterdam UMC, Location University of Amsterdam, Amsterdam, the Netherlands; b Cancer Center Amsterdam, Treatment and Quality of Life, Amsterdam, the Netherlands; c Department of Medical Oncology, Leiden University Medical Centre, Leiden, the Netherlands; d CZ Health Insurance, Tilburg, the Netherlands; e Department of Internal Medicine, Amphia Hospital, Breda, the Netherlands

**Keywords:** Neoplasms, Cancer survivors, Medical overuse, Health care reform, Value-based health care

## Abstract

Cancer care often operates under the assumption that ‘more is better’, resulting in frequent testing, intensive treatments, and constant follow-up. However, this approach has unintended consequences and places significant burdens on patients, providers, and healthcare systems. At the same time, cancer care is evolving rapidly and the number of cancer survivors continues to grow, highlighting the urgent need for reform.

In the Netherlands, important efforts are taking place to de-escalate and de-implement ineffective practices across the cancer care continuum, from screening to palliative care. The purpose of this viewpoint is to highlight these efforts and stimulate reflection across disciplines. Building on these examples, we propose practical strategies for all stakeholders, from patients to payers and policymakers, to help reduce overuse and prioritise value over volume. Through this work, we challenge the existing paradigm and advocate for a shift toward a ‘less is more’ approach in cancer care.

**Funding:**

This viewpoint received no funding.

## Introduction

In recent years, the number of cancer survivors (those living with and beyond cancer) has risen significantly, driven by an ageing population and advances in early detection and treatment.^1^ Innovations, such as immune checkpoint inhibitors (ICIs), have brought major advances in clinical outcomes, but they also come with uncertainties about how to integrate them into clinical practice. Particularly in cancer care, the assumption that ‘more is better’ often results in frequent testing, intensive treatments, and long term follow-up.^2^^,^^3^ However, this approach has unintended consequences, including unnecessary procedures, side effects, increased burdens on hospitals and staff, high costs, and environmental harm.

**Fig. 1:**
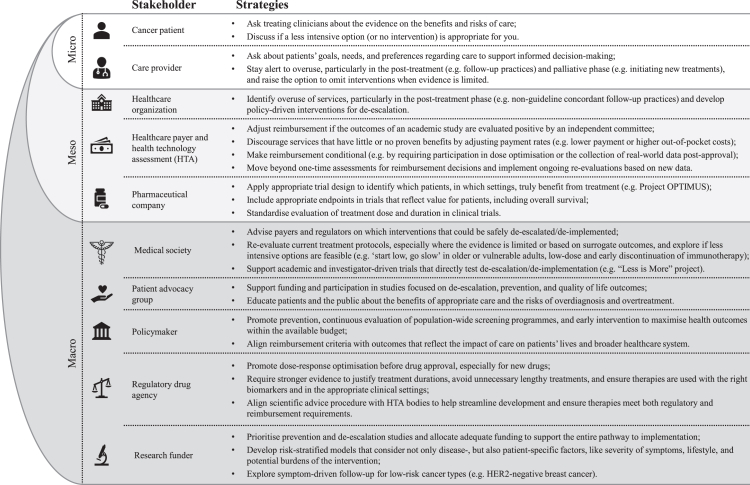
Strategies to reduce overuse in cancer care, by healthcare system level.

As the number of cancer survivors continues to grow, it is increasingly important to prioritise the value of cancer care over volume. Although the concept of value-based care is not new (see Box 1 for key terms), progress has been slow. Most efforts take place at individual departments or hospitals rather than across healthcare systems.^4^^,^^5^ However, in the Netherlands interest in value-based care has recently renewed. One important example is the “Less is More” project, which began with reducing surveillance in low-risk Barett's esophagus and is now expanded to eliminate 13 other ineffective but entrenched medical practices.^6^ This ‘less is more’ approach is part of a broader movement in the Netherlands, with successful examples across the cancer care continuum. The purpose of this viewpoint is not only to highlight these efforts, but also to stimulate reflection across disciplines in daily clinical practice, to challenge assumptions, and to encourage systematic change (Fig. 1). Such an approach must be prioritised continuously and across all levels of healthcare, from policy and funding to clinical practice and patient engagement, to have the best overall impact.

Box 1 **Key terms**

**Table:** 

De-escalation	Reducing the intensity or frequency of interventions when evidence shows they are unnecessary or may cause harm.
De-implementation	Stopping or removing ineffective or low-value care.
Evidence-based practice	Making decisions based on the best available evidence.
Low-value care	Interventions that provide little or no benefit, sometimes with harm.
Overuse	Interventions that are unnecessary, or more than needed, often leading to potential harm, waste, and inefficiency (e.g. overscreening, overdiagnosis, overtreatment).
Value-based care	Improving outcomes that matter to patients relative to costs and recourse use.

## Screening and prevention

Population-wide cancer screening has been debated for decades, especially as access to screening procedures has expanded greatly. While screening can reduce cancer-related mortality in certain high-risk populations, it also has important drawbacks, including high costs, false positive test results, and overdiagnosis of slow-growing tumours that are unlikely to cause harm during the patient's lifetime. In breast cancer, for example, studies suggest that up to half of the screen-detected cancers may be overdiagnosed.^7^ In the Netherlands, the discussion around population-wide screening has gained renewed attention following the publication of a new report by the Council for Public Health and Society.^8^ The report highlights several factors contributing to the controversy, including limited evidence for screening and differing interpretations of survival and mortality statistics. The so-called “popularity paradox” also plays a role: screening typically leads to more diagnoses and therefore appears more valuable, which in turn drives further screening. The report concludes that the benefits of screening are generally presented too optimistically, while the disadvantages, such as overdiagnosis and overtreatment, are overlooked. One example is the current debate about introducing lung cancer screening. While such screening reduces lung cancer-specific mortality, all-cause mortality at 10 years of follow-up remains unchanged.^9^ This may be largely explained by smoking, whose harmful effects go far beyond lung cancer alone. Without addressing these underlying risk factors, the benefits of screening can be limited. Furthermore, this screening poses ethical dilemmas. For instance, if current or former smokers are eligible for lung cancer screening, should passive smokers also be included? The introduction of a lung cancer screening program would place additional pressure on the healthcare system, potentially causing displacement effects and reducing access for other patients. The Council therefore emphasises the need to shift the focus from screening to prevention.^8^ Up to 40% of cancer cases and 44% of cancer deaths are linked to risk factors we can potentially change.^10^ Turning this knowledge into effective prevention strategies may have a large impact on global cancer burden. Nevertheless, healthcare policy makers and research funders tend to prioritise treatment over prevention, highlighting the need for change at the macro-level (Fig. 1).

## Treatment

The high cost of cancer care is largely driven by treatment costs.^11^ Higher costs are justified when treatments deliver substantial clinical benefit, such as ICIs. However, most cancer drugs are approved at the maximum tolerated dose (MTD) established in clinical trials, based on the assumption that higher doses (‘more’) provide greater efficacy (‘is better’).^2^ To speed up access to new treatments, MTDs often become standard label doses without rigorous dose–response optimisation and treatment durations will be lengthy without sufficient justification. Once approved, doses and treatment duration are rarely adjusted. However research showed that 65% of newly registered cancer drugs could benefit from dose–response optimisation, either by dose reduction (32%) or regimen adjustments (32%).^12^

While higher doses are generally more effective for chemotherapies, where killing more cells improves outcomes, it is less certain for newer treatments, like ICIs. Studies showed that ICIs are often given at higher doses than necessary.^13^ In lung cancer, this led to the development of alternative dosing protocols (lower and weight-based) that are more cost-effective.^14^ These alternative protocols also have important environmental benefits, cutting carbon emissions by up to 25%.^15^ Several trials following this ‘less is more’ principle are now underway in the Netherlands, such as the Dedication-1 trial, which compares reduced-dose pembrolizumab with the standard dose in non-small cell lung cancer.^16^ Other trials are looking at shorter treatment durations or treatment-free intervals, such as Apa/Enza Short in metastatic hormone-sensitive prostate cancer, AVE-Short in advanced urothelial carcinoma, FABULOUS (HOVON 174) in multiple myeloma, and the Safe Stop and Safe Stop IPI-NIVO trials in metastatic melanoma.^17^ Recognising the uncertainties around new cancer treatments, regulatory agencies like the Food and Drug Administration (FDA) and European Medicines Agency (EMA) are now encouraging dose–response optimisation before a drug is approved, through initiatives such as Project OPTIMUS.^18^ For drugs that are already on the market, dose reduction trials (DRTs) offer an important alternative. To generate this robust evidence, funders should prioritise pragmatic trials and de-escalation studies that deliver evidence fit for implementation (Fig. 1). Healthcare payers can further promote this knowledge development by making reimbursement conditional, for example by requiring participation in optimised dosing or treatment duration trials, or the collection of real-world data post-approval. A leading example of this approach is the Treatmeds Foundation, established by healthcare payers in the Netherlands, which funds trials that study less intensive treatments across all indications.

Beyond drug therapy, reducing the intensity of other treatment modalities can also be beneficial.^19^ For instance, shorter and lower-dose radiation (‘less’) in breast cancer provides excellent cancer control and better aesthetic outcomes compared to longer treatments (‘is more’).^20^ These findings challenge the assumption that aggressive treatments are always better and encourage medical societies to re-evaluate current treatment protocols, especially when the supporting evidence is limited (Fig. 1). Furthermore, much of the available evidence still relies on surrogate outcomes, such as progression-free survival (PFS), while improvements in clinical outcomes, such overall survival (OS) and quality of life (QOL), remain relatively uncommon.^21^ Only recently, the FDA released draft guidance requiring all randomised oncology trials to assess OS.^22^ This guidance highlights the need for appropriate outcome measures that emphasise the impact on patients' lives, as well as on the broader healthcare system. Defining and achieving these meaningful outcomes requires close collaboration among stakeholders at all levels, along with ongoing monitoring and re-evaluation of new evidence (Fig. 1). In the Netherlands, the Integral Care Agreement was signed in 2022 between medical societies, patient organisations, health insurers, and government. One of its objectives is to periodically re-evaluate the reimbursement of expensive medicines based on new evidence, ensuring that the healthcare budget is not spent on low-value care. A recent example is the re-evaluation of PARP inhibitors across several indications. This re-evaluation, based on new clinical data, has led to the discontinuation of coverage for the non-BRCA tumours due to uncertain survival advantage. It is important to note that these re-evaluations are not solely aimed at cost saving. Savings in one area can free up resources for investment elsewhere, as illustrated in the Dedication-1 trial, where savings are partly reinvested in biomarker research to improve the personalisation of pembrolizumab treatment.^16^

## Post-treatment

Follow-up consists of both routine surveillance for recurrences, as well as assessment and management of treatment related sequelae. It is often assumed that frequent follow-up (‘more’) leads to better cancer outcomes (‘is better’). This assumption has led to the implementation of intensive follow-up schedules, which are often inconsistent, non-specific, and not supported by strong evidence.^23^ The schedules are typically based on clinical trials where surveillance serves a clear purpose, such as measuring surrogate outcomes like PFS. However, in everyday practice, these outcomes may be less relevant. One example is the use of screening MRI of the brain in the adjuvant setting for stage III melanoma. MRIs were performed in clinical trials to monitor disease progression, but their benefit in clinical practice remained unclear. A Dutch cohort study showed that the yield of screening MRI in these patients is low, leading to the de-implementation of this practice.^24^ In fact, reduced surveillance (through fewer visits, examinations, and tests) does not seem to compromise survival outcomes across different cancers.^25^ Many recurrences are diagnosed between scheduled visits when survivors develop symptoms. For some low-risk tumours, a symptom-based approach could therefore be just as effective as routine surveillance. For instance, a large observational study on HER2-negative breast cancer found no survival difference between symptom-detected and routine-detected recurrences.^26^ In such cases, patients may no longer need routine surveillance. Instead, they could be advised to contact an assigned caregiver, such as their general practitioner, if they notice any symptoms. However, this approach raises ethical dilemmas similar to those seen in population-wide screening. Do we dare to screen low-risk patients less often, and how should we respond if cancer is still detected in these patients? On an individual level, the impact of a later recurrence diagnosis may be substantial, but this must be balanced against the large number of visits and tests required for the whole population.^8^

In addition to detection of recurrences, follow-up is aimed at addressing the long-term and late effects of cancer and its treatment. One concern is that reducing follow-up limits the opportunities to address survivors' needs. However, increasing evidence shows that reduced follow-up does not negatively impact patient-reported outcomes, including QOL.^27^ Frequent follow-up may in fact increase anxiety and stress, rather than provide reassurance. In a recent trial from the Netherlands, fewer follow-up visits (‘less’) after low-risk endometrial cancer were associated with higher patient satisfaction (‘is more’).^28^ Currently, most follow-up schedules are designed to detect cancer recurrence, but other factors should also be considered, such as physical symptoms, psychosocial concerns, and the burden of follow-up itself. Further research is needed to understand which follow-up components truly benefit survivors and whether a reduced or symptom-driven approach could work for some cancers.

In recent years, international educational campaigns, such as Choosing Wisely, have tried to raise awareness about ineffective cancer care practices, but progress has been slow, mainly due to established routines of providers and resistance to change.^5^ This resistance is further reinforced by financial and legal considerations impacting specialists. Financial incentives, such as fee-for-service payments, can encourage overuse.^3^ Healthcare payers therefore play an important role by adjusting payment rates for services that have little or no proven benefit (e.g. lower payment or higher out-of-pocket costs) and moving beyond one-time assessments for reimbursement decisions. Additionally, the threat of litigation can lead to ‘defensive medicine’, in which providers recommend more intensive interventions to minimise malpractice risk, even when the potential benefits are small.^3^ This complex interplay of factors highlights the need for a multi-level approach, by educating patients and the public about appropriate care, and identifying the areas of overuse at the institutional level (Fig. 1). Every de-escalation/de-implementation initiative needs to be evidence-based, with appropriate monitoring to evaluate outcomes and safeguard against undertreatment. Traditionally, the clinical guidelines are only revised after research has been conducted and outcomes have been published. However, this process can take years. In the Netherlands, these challenges led to the launch of the “Less is More” project.^6^ This project takes a unique approach to de-escalation/de-implementation by having medical professional organisations identify practices they consider ineffective, but still standard, and by immediately stopping these practices while closely monitoring the effects. In this way, the evidence follows de-implementation rather than the other way around.

## Palliative

As treatment goals shift from cure to palliation, the ‘less is more’ approach can become more relevant, optimising survival time while preserving QOL. For most patients QOL is more important than simply living longer.^29^ However, many patients still overestimate the benefits of palliative treatment, including tumour response, symptom relief, and survival gain.^30^ About 1 in 5 patients even believe that the treatment can cure their disease. These misunderstandings contribute to the frequent use of high-intensity cancer care near the end of life.^31^ Psychological and cultural factors also play an important role here. In many societies, death is perceived as a defeat or failure, resulting in a preference for treatment rather than a focus on palliative care.^3^ This is reflected in expressions such as ‘fighting’ and ‘beating’ cancer. Such narratives can stigmatise palliative care as ‘giving up’ rather than recognising it as appropriate care. Many care providers also feel the need to pursue every possible treatment option, often referred to as the ‘therapeutic imperative’. Clear communication about the treatment goals, benefits, and risks of treatment is important to reduce misunderstandings and support informed-decision making (Fig. 1). However, for such dialogue to take place, more time is needed, for which no funding is currently available.

Both patients and clinicians agree that aggressive treatment is not always more effective, and both are open to discussing more flexible options. For example, in advanced breast cancer, delaying the start of CDK4/6 inhibitors from the first line of therapy to the second line did not affect survival, but significantly reduced the side effects and costs, demonstrating that some treatments can be safely postponed.^32^ Equally important is knowing when to stop treatment. Common reasons include disease progression, severe side effects, or patient preference. However, there is no clear agreement on the right time to stop. With newer treatments, such as ICIs, it may be reasonable to stop earlier, particularly for patients who respond well. Several trial are now conducted in the Netherlands to examine early discontinuation of ICIs, such as the Safe Stop and Safe Stop IPI-NIVO trials in metastatic melanoma.^17^ To help address such questions, along with other uncertainties related to optimal drug combinations, dosing, and patient selection, the Cancer Medicines Forum (CMF) was established by EMA and the European Organisation for Research and Treatment of Cancer (EORTC) to facilitate collaboration between academic researchers, regulators, payers and other stakeholders aimed at improving the use of cancer medicines after market approval.

## Conclusion

High-intensity cancer care has become the norm, but here we advocate that this needs to change. Researchers are identifying areas of overuse and are developing evidence-based solutions for de-escalation. Across the cancer care continuum, significant opportunities exist to reduce unnecessary screening, treatment, and follow-up, without compromising outcomes. In this viewpoint, we have discussed successful examples from the Netherlands where less is more and we outlined strategies for different stakeholders to help reduce overuse (Fig. 1). At the same time, a multi-level approach is needed to create urgency, initiate change, and ensure lasting and maximum benefits throughout the healthcare system. At the micro-level, this means raising awareness of overuse among patients and care providers, and supporting clear communication about the benefits, risks, and goals of care. At the meso- and macro-levels, it requires realigning system incentives to prioritise value over volume and to perform regular re-evaluations that reduce investments in low-value interventions. Initiatives like the “Less is More” project illustrate how close collaboration between key stakeholders can lead to better use of resources and more appropriate care.

## Contributors

Conceptualisation: all authors. Investigation: all authors. Accessed and verified: all authors. Writing original draft: Julien Vos. Writing review & editing: all authors. Visualisation: all authors.

## Declaration of interests

Sahar Barjesteh van Waalwijk van Doorn-Khosrovani reports a grant from the EU Cancer Mission for the PRIME-ROSE project (grant no. 101104269), and received travel support for presentations from the Centre for Innovation in Regulatory Science, Precision Medicine Forum, Cancer Drug Development Forum, Partners for Patients NGO, OMICO, EMA, EP PERMED, and ISPOR.

## Appendix B Supplementary data

Translated Abstract
