# Supplementary material for: Less is more: inverting the paradigm across the cancer care continuum
Source: eClinicalMedicine. 2025 Oct 24;90:103599. doi: 10.1016/j.eclinm.2025.103599 (PMC12593689; doi:10.1016/j.eclinm.2025.103599)
Supplement: Translated Abstract [file mmc1.docx]

The following translations in Dutch were submitted by the authors and we reproduce them as supplied. They have not been peer reviewed. Our editorial processes have only been applied to the original abstract in English, which should serve as reference for this manuscript

**Samenvatting**

In de zorg voor mensen met kanker leeft vaak de gedachte dat ‘meer beter is’, wat leidt tot veel testen, intensieve behandelingen en langdurige controles. Deze gedachte heeft echter onbedoelde gevolgen en legt een grote druk op patiënten, zorgverleners en het zorgsysteem als geheel. Tegelijkertijd verandert de kankerzorg snel en groeit het aantal mensen dat de ziekte overleeft, wat de noodzaak van een nieuwe aanpak benadrukt.

In Nederland zijn verschillende initiatieven gestart om ineffectieve praktijken in de kankerzorg te de-escaleren en te de-implementeren, van screening tot palliatieve zorg. Met dit artikel willen we deze initiatieven uitlichten en reflectie stimuleren binnen verschillende zorgdisciplines. Aan de hand van de voorbeelden doen we praktische voorstellen voor alle betrokken partijen, van patiënten tot zorgverzekeraars en beleidsmakers, om overmatige zorg te verminderen en doelmatige zorg te stimuleren. Met dit werk willen we het bestaande paradigma ter discussie stellen en pleiten we voor een verschuiving naar een ‘less is more’ benadering in de kankerzorg.
